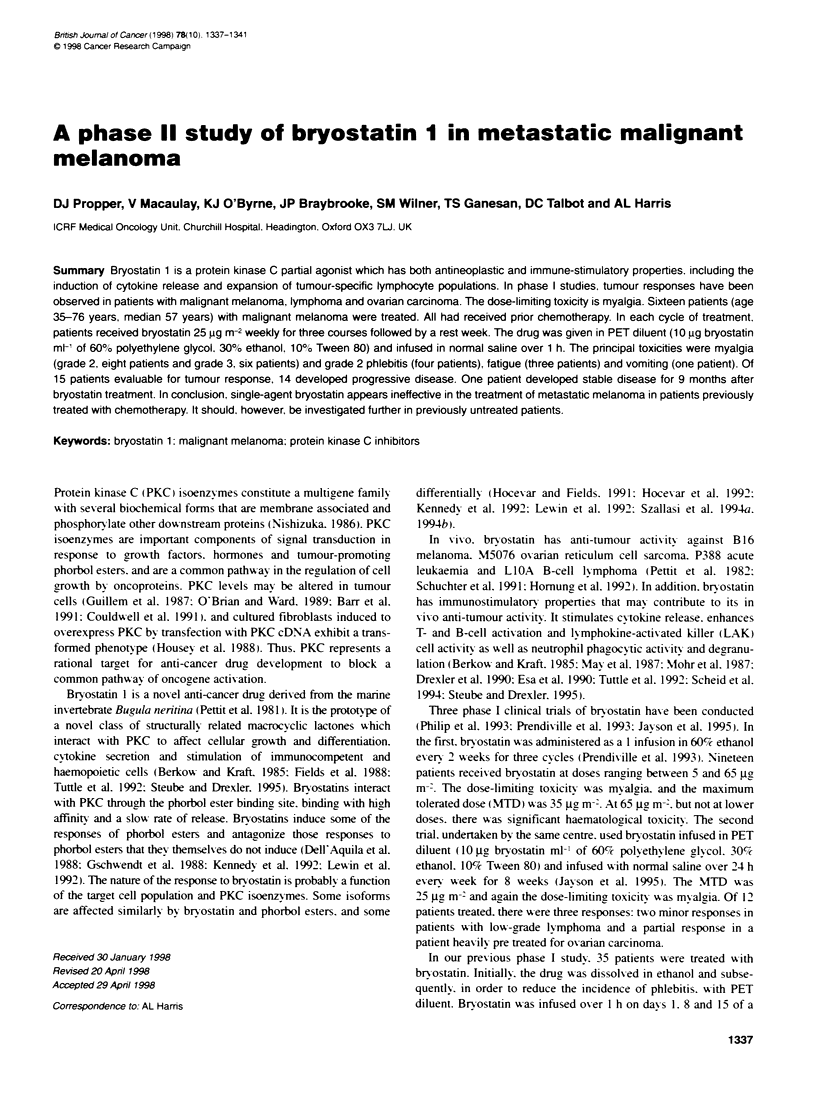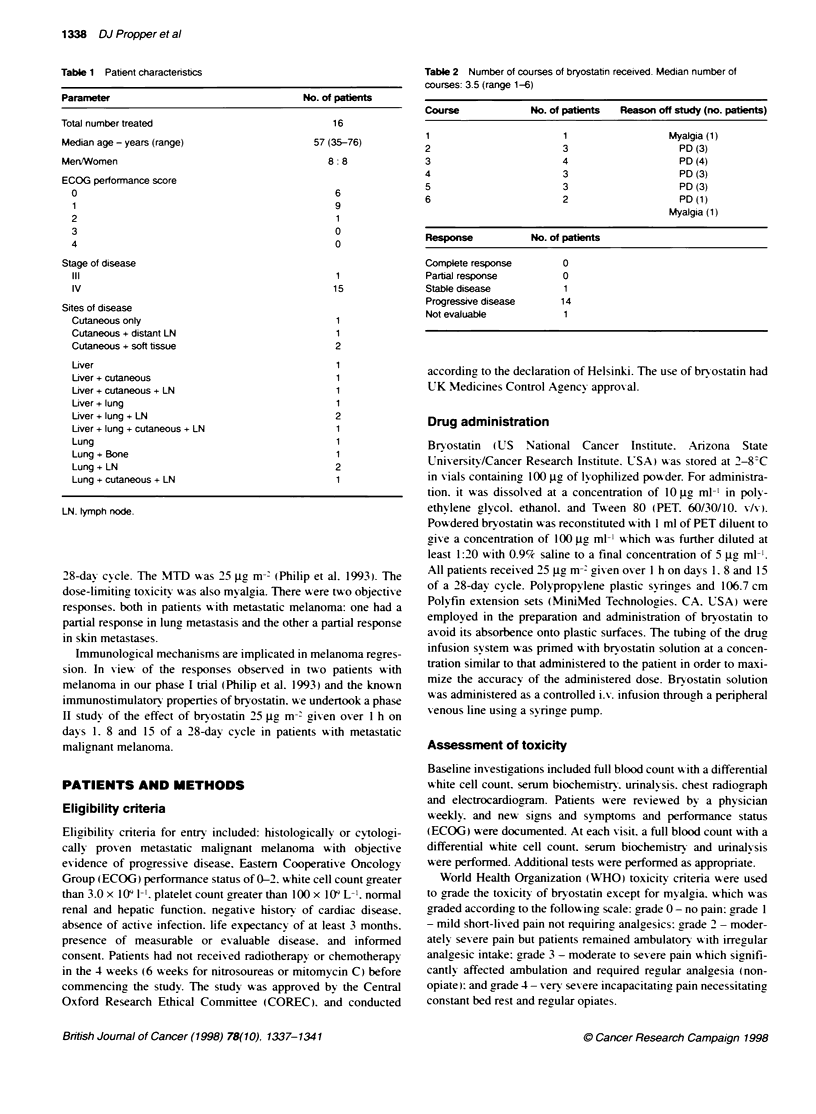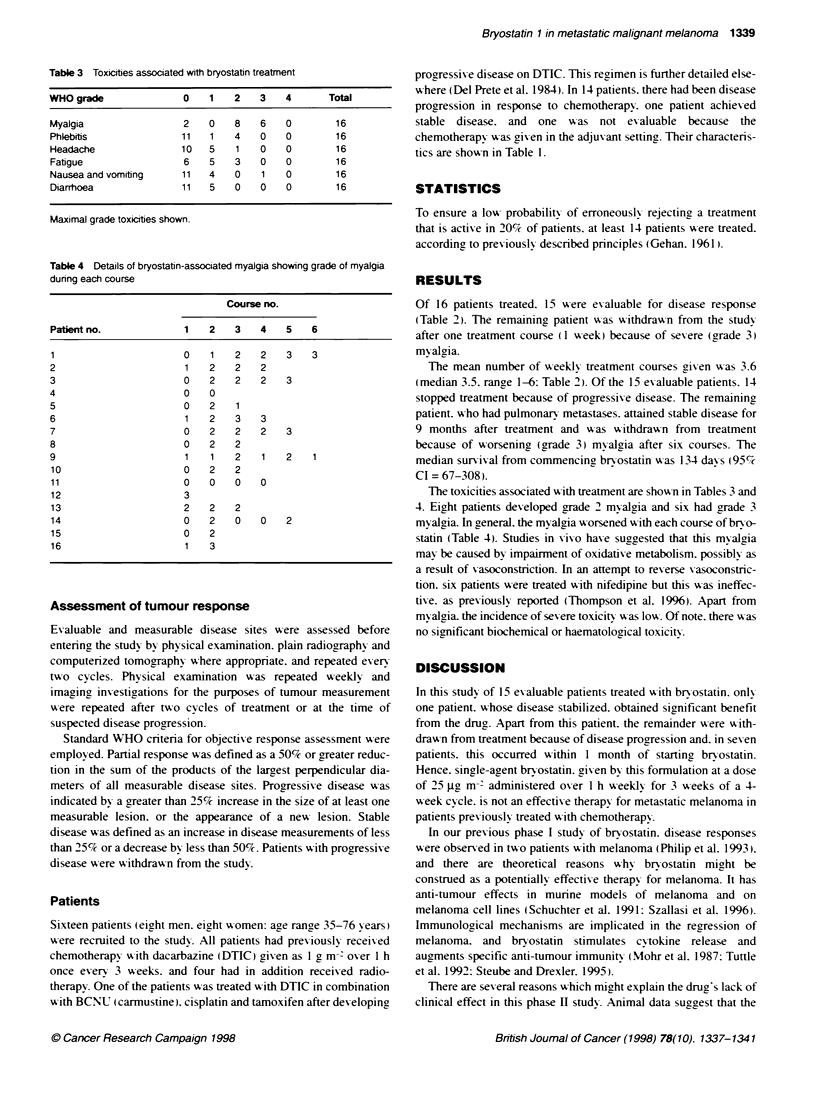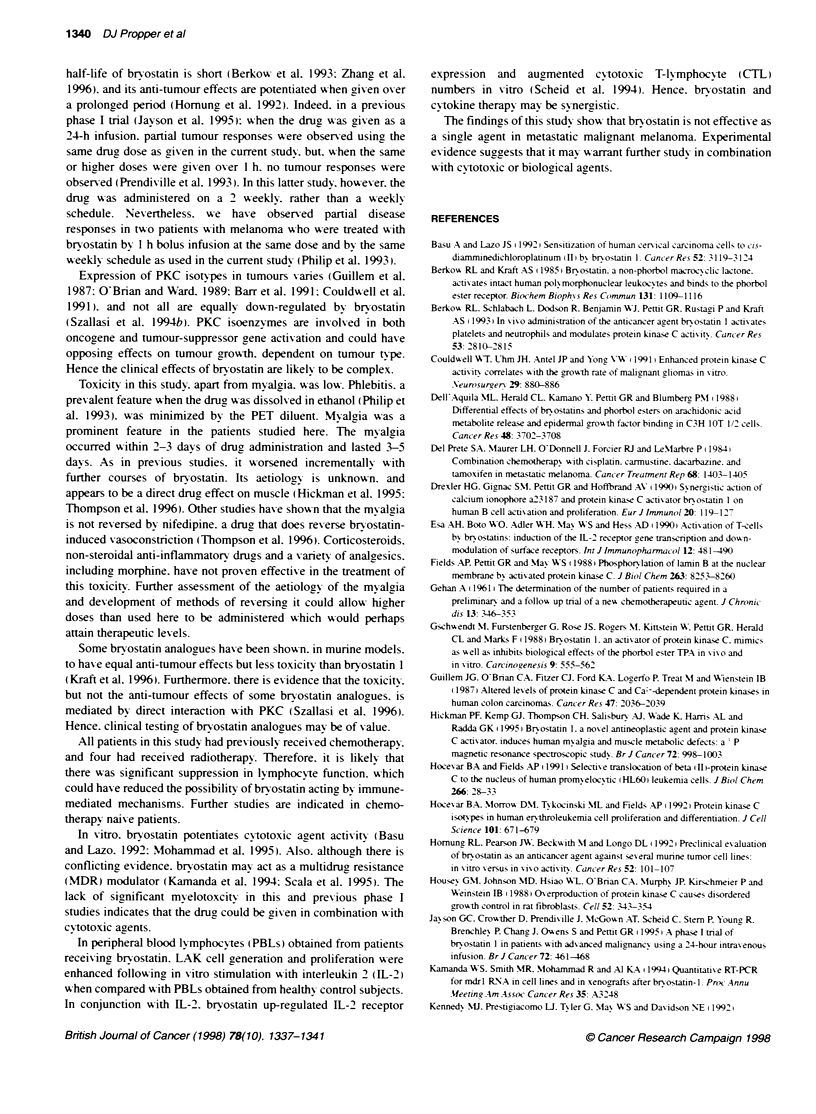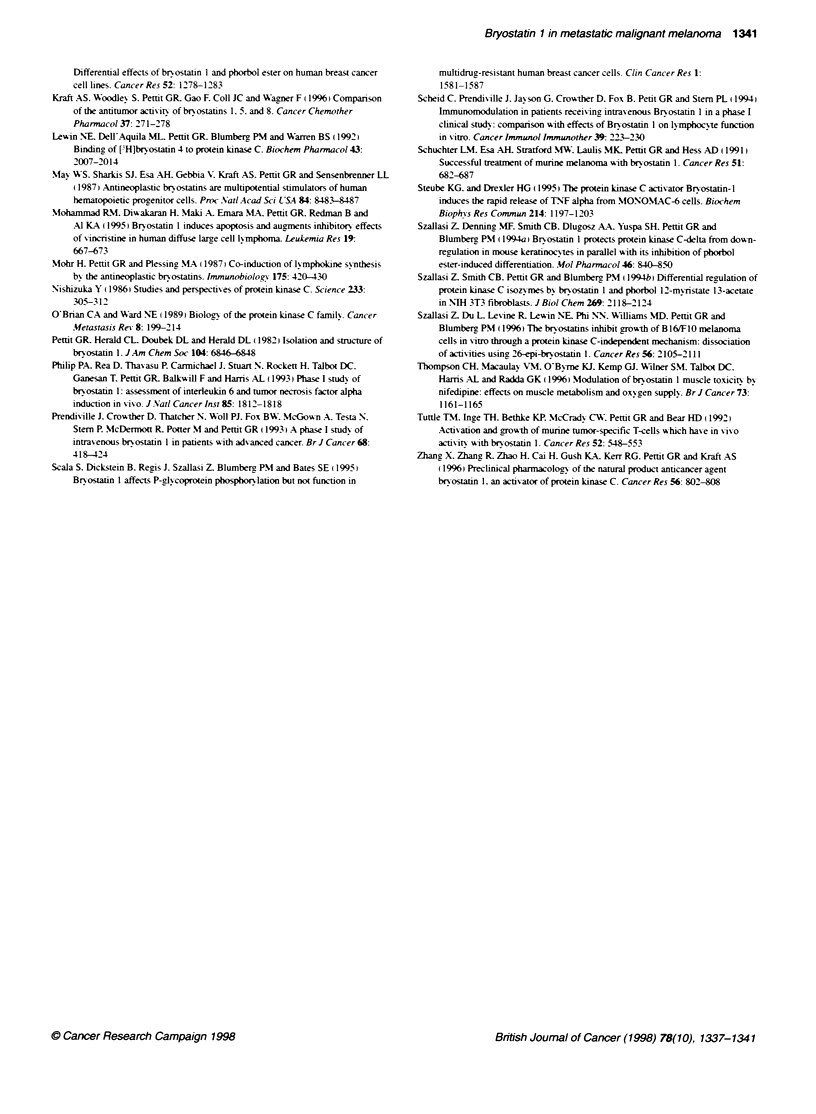# A phase II study of bryostatin 1 in metastatic malignant melanoma.

**DOI:** 10.1038/bjc.1998.680

**Published:** 1998-11

**Authors:** D. J. Propper, V. Macaulay, K. J. O'Byrne, J. P. Braybrooke, S. M. Wilner, T. S. Ganesan, D. C. Talbot, A. L. Harris

**Affiliations:** ICRF Medical Oncology Unit, Churchill Hospital, Headington, Oxford, UK.

## Abstract

Bryostatin 1 is a protein kinase C partial agonist which has both antineoplastic and immune-stimulatory properties, including the induction of cytokine release and expansion of tumour-specific lymphocyte populations. In phase I studies, tumour responses have been observed in patients with malignant melanoma, lymphoma and ovarian carcinoma. The dose-limiting toxicity is myalgia. Sixteen patients (age 35-76 years, median 57 years) with malignant melanoma were treated. All had received prior chemotherapy. In each cycle of treatment, patients received bryostatin 25 degrees g m(-2) weekly for three courses followed by a rest week. The drug was given in PET diluent (10 microg bryostatin ml(-1) of 60% polyethylene glycol, 30% ethanol, 10% Tween 80) and infused in normal saline over 1 h. The principal toxicities were myalgia (grade 2, eight patients and grade 3, six patients) and grade 2 phlebitis (four patients), fatigue (three patients) and vomiting (one patient). Of 15 patients evaluable for tumour response, 14 developed progressive disease. One patient developed stable disease for 9 months after bryostatin treatment. In conclusion, single-agent bryostatin appears ineffective in the treatment of metastatic melanoma in patients previously treated with chemotherapy. It should, however, be investigated further in previously untreated patients.


British Journal of Cancer (1998) 78(10). 1337–1341
© 1998 Cancer Research Campaign

### A phase II study of bryostatin 1 in metastatic malignant melanoma

**DJ Propper, V Macaulay, KJ O'Byrne, JP Braybrooke, SM Wilner, TS Ganesan, DC Talbot and AL Harris**

ICRF Medical Oncology Unit. Churchill Hospital. Headington. Oxford OX3 7LJ. UK

**Summary** Bryostatin 1 is a protein kinase C partial agonist which has both antineoplastic and immune-stimulatory properties. including the induction of cytokine release and expansion of tumour-specific lymphocyte populations. In phase I studies. tumour responses have been observed in patients with malignant melanoma, lymphoma and ovarian carcinoma. The dose-limiting toxicity is myalgia. Sixteen patients (age 35–76 years. median 57 years) with malignant melanoma were treated. All had received prior chemotherapy. In each cycle of treatment. patients received bryostatin 25 μg m$^{-2}$ weekly for three courses followed by a rest week. The drug was given in PET diluent (10 μg bryostatin ml$^{-1}$ of 60% polyethylene glycol. 30% ethanol. 10% Tween 80) and infused in normal saline over 1 h. The principal toxicities were myalgia (grade 2. eight patients and grade 3. six patients) and grade 2 phlebitis (four patients). fatigue (three patients) and vomiting (one patient). Of 15 patients evaluable for tumour response. 14 developed progressive disease. One patient developed stable disease for 9 months after bryostatin treatment. In conclusion. single-agent bryostatin appears ineffective in the treatment of metastatic melanoma in patients previously treated with chemotherapy. It should. however. be investigated further in previously untreated patients.

**Keywords:** bryostatin 1: malignant melanoma: protein kinase C inhibitors

Protein kinase C (PKC) isoenzymes constitute a multigene family with several biochemical forms that are membrane associated and phosphorylate other downstream proteins (Nishizuka. 1986). PKC isoenzymes are important components of signal transduction in response to growth factors. hormones and tumour-promoting phorbol esters. and are a common pathway in the regulation of cell growth by oncoproteins. PKC levels may be altered in tumour cells (Guillem et al. 1987: O'Brian and Ward. 1989: Barr et al. 1991: Couldwell et al. 1991). and cultured fibroblasts induced to overexpress PKC by transfection with PKC cDNA exhibit a transformed phenotype (Housey et al. 1988). Thus. PKC represents a rational target for anti-cancer drug development to block a common pathway of oncogene activation.

Bryostatin 1 is a novel anti-cancer drug derived from the marine invertebrate *Bugula neritina* (Pettit et al. 1981). It is the prototype of a novel class of structurally related macrocyclic lactones which interact with PKC to affect cellular growth and differentiation. cytokine secretion and stimulation of immunocompetent and haemopoietic cells (Berkow and Kraft. 1985: Fields et al. 1988: Tuttle et al. 1992: Steube and Drexler. 1995). Bryostatins interact with PKC through the phorbol ester binding site. binding with high affinity and a slow rate of release. Bryostatins induce some of the responses of phorbol esters and antagonize those responses to phorbol esters that they themselves do not induce (Dell'Aquila et al. 1988: Gschwendt et al. 1988: Kennedy et al. 1992: Lewin et al. 1992). The nature of the response to bryostatin is probably a function of the target cell population and PKC isoenzymes. Some isoforms are affected similarly by bryostatin and phorbol esters. and some differentially (Hocevar and Fields. 1991: Hocevar et al. 1992: Kennedy et al. 1992: Lewin et al. 1992: Szallasi et al. 1994*a*. 1994*b*).

In vivo. bryostatin has anti-tumour activity against B16 melanoma. M5076 ovarian reticulum cell sarcoma. P388 acute leukaemia and L10A B-cell lymphoma (Pettit et al. 1982: Schuchter et al. 1991: Hornung et al. 1992). In addition. bryostatin has immunostimulatory properties that may contribute to its in vivo anti-tumour activity. It stimulates cytokine release. enhances T- and B-cell activation and lymphokine-activated killer (LAK) cell activity as well as neutrophil phagocytic activity and degranulation (Berkow and Kraft. 1985: May et al. 1987: Mohr et al. 1987: Drexler et al. 1990: Esa et al. 1990: Tuttle et al. 1992: Scheid et al. 1994: Steube and Drexler. 1995).

Three phase I clinical trials of bryostatin have been conducted (Philip et al. 1993: Prendiville et al. 1993: Jayson et al. 1995). In the first. bryostatin was administered as a 1 infusion in 60% ethanol every 2 weeks for three cycles (Prendiville et al. 1993). Nineteen patients received bryostatin at doses ranging between 5 and 65 μg m$^{-2}$. The dose-limiting toxicity was myalgia. and the maximum tolerated dose (MTD) was 35 μg m$^{-2}$. At 65 μg m$^{-2}$. but not at lower doses. there was significant haematological toxicity. The second trial. undertaken by the same centre. used bryostatin infused in PET diluent (10 μg bryostatin ml$^{-1}$ of 60% polyethylene glycol. 30% ethanol. 10% Tween 80) and infused with normal saline over 24 h every week for 8 weeks (Jayson et al. 1995). The MTD was 25 μg m$^{-2}$ and again the dose-limiting toxicity was myalgia. Of 12 patients treated. there were three responses: two minor responses in patients with low-grade lymphoma and a partial response in a patient heavily pre treated for ovarian carcinoma.

In our previous phase I study. 35 patients were treated with bryostatin. Initially. the drug was dissolved in ethanol and subsequently. in order to reduce the incidence of phlebitis. with PET diluent. Bryostatin was infused over 1 h on days 1. 8 and 15 of a

*Received 30 January 1998*
*Revised 20 April 1998*
*Accepted 29 April 1998*

*Correspondence to:* AL Harris

**Table 1** Patient characteristics

| Parameter | No. of patients |
|---|---|
| Total number treated | 16 |
| Median age – years (range) | 57 (35–76) |
| Men/Women | 8 : 8 |
| ECOG performance score | |
| 0 | 6 |
| 1 | 9 |
| 2 | 1 |
| 3 | 0 |
| 4 | 0 |
| Stage of disease | |
| III | 1 |
| IV | 15 |
| Sites of disease | |
| Cutaneous only | 1 |
| Cutaneous + distant LN | 1 |
| Cutaneous + soft tissue | 2 |
| Liver | 1 |
| Liver + cutaneous | 1 |
| Liver + cutaneous + LN | 1 |
| Liver + lung | 1 |
| Liver + lung + LN | 2 |
| Liver + lung + cutaneous + LN | 1 |
| Lung | 1 |
| Lung + Bone | 1 |
| Lung + LN | 2 |
| Lung + cutaneous + LN | 1 |

LN, lymph node.

**Table 2** Number of courses of bryostatin received. Median number of courses: 3.5 (range 1–6)

| Course | No. of patients | Reason off study (no. patients) |
|---|---|---|
| 1 | 1 | Myalgia (1) |
| 2 | 3 | PD (3) |
| 3 | 4 | PD (4) |
| 4 | 3 | PD (3) |
| 5 | 3 | PD (3) |
| 6 | 2 | PD (1)<br>Myalgia (1) |

| Response | No. of patients |
|---|---|
| Complete response | 0 |
| Partial response | 0 |
| Stable disease | 1 |
| Progressive disease | 14 |
| Not evaluable | 1 |

28-day cycle. The MTD was 25 μg m$^{-2}$ (Philip et al, 1993). The dose-limiting toxicity was also myalgia. There were two objective responses, both in patients with metastatic melanoma: one had a partial response in lung metastasis and the other a partial response in skin metastases.

Immunological mechanisms are implicated in melanoma regression. In view of the responses observed in two patients with melanoma in our phase I trial (Philip et al, 1993) and the known immunostimulatory properties of bryostatin, we undertook a phase II study of the effect of bryostatin 25 μg m$^{-2}$ given over 1 h on days 1, 8 and 15 of a 28-day cycle in patients with metastatic malignant melanoma.

### PATIENTS AND METHODS

### Eligibility criteria

Eligibility criteria for entry included: histologically or cytologically proven metastatic malignant melanoma with objective evidence of progressive disease, Eastern Cooperative Oncology Group (ECOG) performance status of 0–2, white cell count greater than 3.0 × 10$^9$ l$^{-1}$, platelet count greater than 100 × 10$^9$ L$^{-1}$, normal renal and hepatic function, negative history of cardiac disease, absence of active infection, life expectancy of at least 3 months, presence of measurable or evaluable disease, and informed consent. Patients had not received radiotherapy or chemotherapy in the 4 weeks (6 weeks for nitrosoureas or mitomycin C) before commencing the study. The study was approved by the Central Oxford Research Ethical Committee (COREC), and conducted according to the declaration of Helsinki. The use of bryostatin had UK Medicines Control Agency approval.

### Drug administration

Bryostatin (US National Cancer Institute, Arizona State University/Cancer Research Institute, USA) was stored at 2–8°C in vials containing 100 μg of lyophilized powder. For administration, it was dissolved at a concentration of 10 μg ml$^{-1}$ in polyethylene glycol, ethanol, and Tween 80 (PET, 60/30/10, v/v). Powdered bryostatin was reconstituted with 1 ml of PET diluent to give a concentration of 100 μg ml$^{-1}$ which was further diluted at least 1:20 with 0.9% saline to a final concentration of 5 μg ml$^{-1}$. All patients received 25 μg m$^{-2}$ given over 1 h on days 1, 8 and 15 of a 28-day cycle. Polypropylene plastic syringes and 106.7 cm Polyfin extension sets (MiniMed Technologies, CA, USA) were employed in the preparation and administration of bryostatin to avoid its absorbence onto plastic surfaces. The tubing of the drug infusion system was primed with bryostatin solution at a concentration similar to that administered to the patient in order to maximize the accuracy of the administered dose. Bryostatin solution was administered as a controlled i.v. infusion through a peripheral venous line using a syringe pump.

### Assessment of toxicity

Baseline investigations included full blood count with a differential white cell count, serum biochemistry, urinalysis, chest radiograph and electrocardiogram. Patients were reviewed by a physician weekly, and new signs and symptoms and performance status (ECOG) were documented. At each visit, a full blood count with a differential white cell count, serum biochemistry and urinalysis were performed. Additional tests were performed as appropriate.

World Health Organization (WHO) toxicity criteria were used to grade the toxicity of bryostatin except for myalgia, which was graded according to the following scale: grade 0 – no pain; grade 1 – mild short-lived pain not requiring analgesics; grade 2 – moderately severe pain but patients remained ambulatory with irregular analgesic intake; grade 3 – moderate to severe pain which significantly affected ambulation and required regular analgesia (non-opiate); and grade 4 – very severe incapacitating pain necessitating constant bed rest and regular opiates.

© Cancer Research Campaign 1998

**Table 3** Toxicities associated with bryostatin treatment

| WHO grade | 0 | 1 | 2 | 3 | 4 | Total |
|---|---|---|---|---|---|---|
| Myalgia | 2 | 0 | 8 | 6 | 0 | 16 |
| Phlebitis | 11 | 1 | 4 | 0 | 0 | 16 |
| Headache | 10 | 5 | 1 | 0 | 0 | 16 |
| Fatigue | 6 | 5 | 3 | 0 | 0 | 16 |
| Nausea and vomiting | 11 | 4 | 0 | 1 | 0 | 16 |
| Diarrhoea | 11 | 5 | 0 | 0 | 0 | 16 |

Maximal grade toxicities shown.

**Table 4** Details of bryostatin-associated myalgia showing grade of myalgia during each course

| | Course no. | | | | | |
|---|---|---|---|---|---|---|
| Patient no. | 1 | 2 | 3 | 4 | 5 | 6 |
| 1 | 0 | 1 | 2 | 2 | 3 | 3 |
| 2 | 1 | 2 | 2 | 2 | | |
| 3 | 0 | 2 | 2 | 2 | 3 | |
| 4 | 0 | 0 | | | | |
| 5 | 0 | 2 | 1 | | | |
| 6 | 1 | 2 | 3 | 3 | | |
| 7 | 0 | 2 | 2 | 2 | 3 | |
| 8 | 0 | 2 | 2 | | | |
| 9 | 1 | 1 | 2 | 1 | 2 | 1 |
| 10 | 0 | 2 | 2 | | | |
| 11 | 0 | 0 | 0 | 0 | | |
| 12 | 3 | | | | | |
| 13 | 2 | 2 | 2 | | | |
| 14 | 0 | 2 | 0 | 0 | 2 | |
| 15 | 0 | 2 | | | | |
| 16 | 1 | 3 | | | | |

### Assessment of tumour response

Evaluable and measurable disease sites were assessed before entering the study by physical examination, plain radiography and computerized tomography where appropriate, and repeated every two cycles. Physical examination was repeated weekly and imaging investigations for the purposes of tumour measurement were repeated after two cycles of treatment or at the time of suspected disease progression.

Standard WHO criteria for objective response assessment were employed. Partial response was defined as a 50% or greater reduction in the sum of the products of the largest perpendicular diameters of all measurable disease sites. Progressive disease was indicated by a greater than 25% increase in the size of at least one measurable lesion, or the appearance of a new lesion. Stable disease was defined as an increase in disease measurements of less than 25% or a decrease by less than 50%. Patients with progressive disease were withdrawn from the study.

### Patients

Sixteen patients (eight men, eight women: age range 35–76 years) were recruited to the study. All patients had previously received chemotherapy with dacarbazine (DTIC) given as 1 g $m^{-2}$ over 1 h once every 3 weeks, and four had in addition received radiotherapy. One of the patients was treated with DTIC in combination with BCNU (carmustine), cisplatin and tamoxifen after developing progressive disease on DTIC. This regimen is further detailed elsewhere (Del Prete et al, 1984). In 14 patients, there had been disease progression in response to chemotherapy, one patient achieved stable disease, and one was not evaluable because the chemotherapy was given in the adjuvant setting. Their characteristics are shown in Table 1.

### STATISTICS

To ensure a low probability of erroneously rejecting a treatment that is active in 20% of patients, at least 14 patients were treated, according to previously described principles (Gehan, 1961).

### RESULTS

Of 16 patients treated, 15 were evaluable for disease response (Table 2). The remaining patient was withdrawn from the study after one treatment course (1 week) because of severe (grade 3) myalgia.

The mean number of weekly treatment courses given was 3.6 (median 3.5, range 1–6: Table 2). Of the 15 evaluable patients, 14 stopped treatment because of progressive disease. The remaining patient, who had pulmonary metastases, attained stable disease for 9 months after treatment and was withdrawn from treatment because of worsening (grade 3) myalgia after six courses. The median survival from commencing bryostatin was 134 days (95% CI = 67–308).

The toxicities associated with treatment are shown in Tables 3 and 4. Eight patients developed grade 2 myalgia and six had grade 3 myalgia. In general, the myalgia worsened with each course of bryostatin (Table 4). Studies in vivo have suggested that this myalgia may be caused by impairment of oxidative metabolism, possibly as a result of vasoconstriction. In an attempt to reverse vasoconstriction, six patients were treated with nifedipine but this was ineffective, as previously reported (Thompson et al, 1996). Apart from myalgia, the incidence of severe toxicity was low. Of note, there was no significant biochemical or haematological toxicity.

### DISCUSSION

In this study of 15 evaluable patients treated with bryostatin, only one patient, whose disease stabilized, obtained significant benefit from the drug. Apart from this patient, the remainder were withdrawn from treatment because of disease progression and, in seven patients, this occurred within 1 month of starting bryostatin. Hence, single-agent bryostatin, given by this formulation at a dose of 25 μg $m^{-2}$ administered over 1 h weekly for 3 weeks of a 4-week cycle, is not an effective therapy for metastatic melanoma in patients previously treated with chemotherapy.

In our previous phase I study of bryostatin, disease responses were observed in two patients with melanoma (Philip et al, 1993), and there are theoretical reasons why bryostatin might be construed as a potentially effective therapy for melanoma. It has anti-tumour effects in murine models of melanoma and on melanoma cell lines (Schuchter et al, 1991; Szallasi et al, 1996). Immunological mechanisms are implicated in the regression of melanoma, and bryostatin stimulates cytokine release and augments specific anti-tumour immunity (Mohr et al, 1987; Tuttle et al, 1992; Steube and Drexler, 1995).

There are several reasons which might explain the drug's lack of clinical effect in this phase II study. Animal data suggest that the

© Cancer Research Campaign 1998

half-life of bryostatin is short (Berkow et al. 1993; Zhang et al. 1996), and its anti-tumour effects are potentiated when given over a prolonged period (Hornung et al. 1992). Indeed, in a previous phase I trial (Jayson et al. 1995): when the drug was given as a 24-h infusion, partial tumour responses were observed using the same drug dose as given in the current study, but, when the same or higher doses were given over 1 h, no tumour responses were observed (Prendiville et al. 1993). In this latter study, however, the drug was administered on a 2 weekly, rather than a weekly schedule. Nevertheless, we have observed partial disease responses in two patients with melanoma who were treated with bryostatin by 1 h bolus infusion at the same dose and by the same weekly schedule as used in the current study (Philip et al. 1993).

Expression of PKC isotypes in tumours varies (Guillem et al. 1987; O'Brian and Ward, 1989; Barr et al. 1991; Couldwell et al. 1991), and not all are equally down-regulated by bryostatin (Szallasi et al. 1994*b*). PKC isoenzymes are involved in both oncogene and tumour-suppressor gene activation and could have opposing effects on tumour growth, dependent on tumour type. Hence the clinical effects of bryostatin are likely to be complex.

Toxicity in this study, apart from myalgia, was low. Phlebitis, a prevalent feature when the drug was dissolved in ethanol (Philip et al. 1993), was minimized by the PET diluent. Myalgia was a prominent feature in the patients studied here. The myalgia occurred within 2–3 days of drug administration and lasted 3–5 days. As in previous studies, it worsened incrementally with further courses of bryostatin. Its aetiology is unknown, and appears to be a direct drug effect on muscle (Hickman et al. 1995; Thompson et al. 1996). Other studies have shown that the myalgia is not reversed by nifedipine, a drug that does reverse bryostatin-induced vasoconstriction (Thompson et al. 1996). Corticosteroids, non-steroidal anti-inflammatory drugs and a variety of analgesics, including morphine, have not proven effective in the treatment of this toxicity. Further assessment of the aetiology of the myalgia and development of methods of reversing it could allow higher doses than used here to be administered which would perhaps attain therapeutic levels.

Some bryostatin analogues have been shown, in murine models, to have equal anti-tumour effects but less toxicity than bryostatin 1 (Kraft et al. 1996). Furthermore, there is evidence that the toxicity, but not the anti-tumour effects of some bryostatin analogues, is mediated by direct interaction with PKC (Szallasi et al. 1996). Hence, clinical testing of bryostatin analogues may be of value.

All patients in this study had previously received chemotherapy, and four had received radiotherapy. Therefore, it is likely that there was significant suppression in lymphocyte function, which could have reduced the possibility of bryostatin acting by immune-mediated mechanisms. Further studies are indicated in chemotherapy naive patients.

In vitro, bryostatin potentiates cytotoxic agent activity (Basu and Lazo, 1992; Mohammad et al. 1995). Also, although there is conflicting evidence, bryostatin may act as a multidrug resistance (MDR) modulator (Kamanda et al. 1994; Scala et al. 1995). The lack of significant myelotoxcity in this and previous phase I studies indicates that the drug could be given in combination with cytotoxic agents.

In peripheral blood lymphocytes (PBLs) obtained from patients receiving bryostatin, LAK cell generation and proliferation were enhanced following in vitro stimulation with interleukin 2 (IL-2) when compared with PBLs obtained from healthy control subjects. In conjunction with IL-2, bryostatin up-regulated IL-2 receptor expression and augmented cytotoxic T-lymphocyte (CTL) numbers in vitro (Scheid et al. 1994). Hence, bryostatin and cytokine therapy may be synergistic.

The findings of this study show that bryostatin is not effective as a single agent in metastatic malignant melanoma. Experimental evidence suggests that it may warrant further study in combination with cytotoxic or biological agents.

### REFERENCES

Basu A and Lazo JS (1992) Sensitization of human cervical carcinoma cells to *cis*-diamminedichloroplatinum (II) by bryostatin 1. *Cancer Res* **52**: 3119–3124

Berkow RL and Kraft AS (1985) Bryostatin, a non-phorbol macrocyclic lactone, activates intact human polymorphonuclear leukocytes and binds to the phorbol ester receptor. *Biochem Biophys Res Commun* **131**: 1109–1116

Berkow RL, Schlabach L, Dodson R, Benjamin WJ, Pettit GR, Rustagi P and Kraft AS (1993) In vivo administration of the anticancer agent bryostatin 1 activates platelets and neutrophils and modulates protein kinase C activity. *Cancer Res* **53**: 2810–2815

Couldwell WT, Uhm JH, Antel JP and Yong VW (1991) Enhanced protein kinase C activity correlates with the growth rate of malignant gliomas in vitro. *Neurosurgery* **29**: 880–886

Dell'Aquila ML, Herald CL, Kamano Y, Pettit GR and Blumberg PM (1988) Differential effects of bryostatins and phorbol esters on arachidonic acid metabolite release and epidermal growth factor binding in C3H 10T 1/2 cells. *Cancer Res* **48**: 3702–3708

Del Prete SA, Maurer LH, O'Donnell J, Forcier RJ and LeMarbre P (1984) Combination chemotherapy with cisplatin, carmustine, dacarbazine, and tamoxifen in metastatic melanoma. *Cancer Treatment Rep* **68**: 1403–1405

Drexler HG, Gignac SM, Pettit GR and Hoffbrand AV (1990) Synergistic action of calcium ionophore a23187 and protein kinase C activator bryostatin 1 on human B cell activation and proliferation. *Eur J Immunol* **20**: 119–127

Esa AH, Boto WO, Adler WH, May WS and Hess AD (1990) Activation of T-cells by bryostatins: induction of the IL-2 receptor gene transcription and down-modulation of surface receptors. *Int J Immunopharmacol* **12**: 481–490

Fields AP, Pettit GR and May WS (1988) Phosphorylation of lamin B at the nuclear membrane by activated protein kinase C. *J Biol Chem* **263**: 8253–8260

Gehan A (1961) The determination of the number of patients required in a preliminary and a follow up trial of a new chemotherapeutic agent. *J Chronic dis* **13**: 346–353

Gschwendt M, Furstenberger G, Rose JS, Rogers M, Kittstein W, Pettit GR, Herald CL and Marks F (1988) Bryostatin 1, an activator of protein kinase C, mimics as well as inhibits biological effects of the phorbol ester TPA in vivo and in vitro. *Carcinogenesis* **9**: 555–562

Guillem JG, O'Brian CA, Fitzer CJ, Ford KA, Logerfo P, Treat M and Wienstein IB (1987) Altered levels of protein kinase C and $Ca^{2+}$-dependent protein kinases in human colon carcinomas. *Cancer Res* **47**: 2036–2039

Hickman PF, Kemp GJ, Thompson CH, Salisbury AJ, Wade K, Harris AL and Radda GK (1995) Bryostatin 1, a novel antineoplastic agent and protein kinase C activator, induces human myalgia and muscle metabolic defects: a $^{31}P$ magnetic resonance spectroscopic study. *Br J Cancer* **72**: 998–1003

Hocevar BA and Fields AP (1991) Selective translocation of beta (II)-protein kinase C to the nucleus of human promyelocytic (HL60) leukemia cells. *J Biol Chem* **266**: 28–33

Hocevar BA, Morrow DM, Tykocinski ML and Fields AP (1992) Protein kinase C isotypes in human erythroleukemia cell proliferation and differentiation. *J Cell Science* **101**: 671–679

Hornung RL, Pearson JW, Beckwith M and Longo DL (1992) Preclinical evaluation of bryostatin as an anticancer agent against several murine tumor cell lines: in vitro versus in vivo activity. *Cancer Res* **52**: 101–107

Housey GM, Johnson MD, Hsiao WL, O'Brian CA, Murphy JP, Kirschmeier P and Weinstein IB (1988) Overproduction of protein kinase C causes disordered growth control in rat fibroblasts. *Cell* **52**: 343–354

Jayson GC, Crowther D, Prendiville J, McGown AT, Scheid C, Stern P, Young R, Brenchley P, Chang J, Owens S and Pettit GR (1995) A phase I trial of bryostatin 1 in patients with advanced malignancy using a 24-hour intravenous infusion. *Br J Cancer* **72**: 461–468

Kamanda WS, Smith MR, Mohammad R and Al KA (1994) Quantitative RT-PCR for mdr1 RNA in cell lines and in xenografts after bryostatin-1. *Proc Annu Meeting Am Assoc Cancer Res* **35**: A3248

Kennedy MJ, Prestigiacomo LJ, Tyler G, May WS and Davidson NE (1992)

© Cancer Research Campaign 1998

Differential effects of bryostatin 1 and phorbol ester on human breast cancer cell lines. *Cancer Res* **52**: 1278–1283

Kraft AS. Woodley S. Pettit GR. Gao F. Coll JC and Wagner F (1996) Comparison of the antitumor activity of bryostatins 1, 5, and 8. *Cancer Chemother Pharmacol* **37**: 271–278

Lewin NE. Dell'Aquila ML. Pettit GR. Blumberg PM and Warren BS (1992) Binding of [$^3$H]bryostatin 4 to protein kinase C. *Biochem Pharmacol* **43**: 2007–2014

May WS. Sharkis SJ. Esa AH. Gebbia V. Kraft AS. Pettit GR and Sensenbrenner LL (1987) Antineoplastic bryostatins are multipotential stimulators of human hematopoietic progenitor cells. *Proc Natl Acad Sci USA* **84**: 8483–8487

Mohammad RM. Diwakaran H. Maki A. Emara MA. Pettit GR. Redman B and Al KA (1995) Bryostatin 1 induces apoptosis and augments inhibitory effects of vincristine in human diffuse large cell lymphoma. *Leukemia Res* **19**: 667–673

Mohr H. Pettit GR and Plessing MA (1987) Co-induction of lymphokine synthesis by the antineoplastic bryostatins. *Immunobiology* **175**: 420–430

Nishizuka Y (1986) Studies and perspectives of protein kinase C. *Science* **233**: 305–312

O'Brian CA and Ward NE (1989) Biology of the protein kinase C family. *Cancer Metastasis Rev* **8**: 199–214

Pettit GR. Herald CL. Doubek DL and Herald DL (1982) Isolation and structure of bryostatin 1. *J Am Chem Soc* **104**: 6846–6848

Philip PA. Rea D. Thavasu P. Carmichael J. Stuart N. Rockett H. Talbot DC. Ganesan T. Pettit GR. Balkwill F and Harris AL (1993) Phase I study of bryostatin 1: assessment of interleukin 6 and tumor necrosis factor alpha induction in vivo. *J Natl Cancer Inst* **85**: 1812–1818

Prendiville J. Crowther D. Thatcher N. Woll PJ. Fox BW. McGown A. Testa N. Stern P. McDermott R. Potter M and Pettit GR (1993) A phase I study of intravenous bryostatin 1 in patients with advanced cancer. *Br J Cancer* **68**: 418–424

Scala S. Dickstein B. Regis J. Szallasi Z. Blumberg PM and Bates SE (1995) Bryostatin 1 affects P-glycoprotein phosphorylation but not function in multidrug-resistant human breast cancer cells. *Clin Cancer Res* **1**: 1581–1587

Scheid C. Prendiville J. Jayson G. Crowther D. Fox B. Pettit GR and Stern PL (1994) Immunomodulation in patients receiving intravenous Bryostatin 1 in a phase I clinical study: comparison with effects of Bryostatin 1 on lymphocyte function in vitro. *Cancer Immunol Immunother* **39**: 223–230

Schuchter LM. Esa AH. Stratford MW. Laulis MK. Pettit GR and Hess AD (1991) Successful treatment of murine melanoma with bryostatin 1. *Cancer Res* **51**: 682–687

Steube KG. and Drexler HG (1995) The protein kinase C activator Bryostatin-1 induces the rapid release of TNF alpha from MONOMAC-6 cells. *Biochem Biophys Res Commun* **214**: 1197–1203

Szallasi Z. Denning MF. Smith CB. Dlugosz AA. Yuspa SH. Pettit GR and Blumberg PM (1994*a*) Bryostatin 1 protects protein kinase C-delta from down-regulation in mouse keratinocytes in parallel with its inhibition of phorbol ester-induced differentiation. *Mol Pharmacol* **46**: 840–850

Szallasi Z. Smith CB. Pettit GR and Blumberg PM (1994*b*) Differential regulation of protein kinase C isozymes by bryostatin 1 and phorbol 12-myristate 13-acetate in NIH 3T3 fibroblasts. *J Biol Chem* **269**: 2118–2124

Szallasi Z. Du L. Levine R. Lewin NE. Phi NN. Williams MD. Pettit GR and Blumberg PM (1996) The bryostatins inhibit growth of B16/F10 melanoma cells in vitro through a protein kinase C-independent mechanism: dissociation of activities using 26-epi-bryostatin 1. *Cancer Res* **56**: 2105–2111

Thompson CH. Macaulay VM. O'Byrne KJ. Kemp GJ. Wilner SM. Talbot DC. Harris AL and Radda GK (1996) Modulation of bryostatin 1 muscle toxicity by nifedipine: effects on muscle metabolism and oxygen supply. *Br J Cancer* **73**: 1161–1165

Tuttle TM. Inge TH. Bethke KP. McCrady CW. Pettit GR and Bear HD (1992) Activation and growth of murine tumor-specific T-cells which have in vivo activity with bryostatin 1. *Cancer Res* **52**: 548–553

Zhang X. Zhang R. Zhao H. Cai H. Gush KA. Kerr RG. Pettit GR and Kraft AS (1996) Preclinical pharmacology of the natural product anticancer agent bryostatin 1, an activator of protein kinase C. *Cancer Res* **56**: 802–808

© Cancer Research Campaign 1998